# Randomized Clinical Trial of Composite Restorations in Primary Teeth: Effect of Adhesive System after Three Years

**DOI:** 10.1155/2016/5409392

**Published:** 2016-10-19

**Authors:** Secil Bektaş Donmez, Melek D. Turgut, Serdar Uysal, Pinar Ozdemir, Meryem Tekcicek, Brigitte Zimmerli, Adrian Lussi

**Affiliations:** ^1^Department of Pediatric Dentistry, Hacettepe University, Ankara, Turkey; ^2^Department of Maxillofacial Radiology, Hacettepe University, Ankara, Turkey; ^3^Department of Biostatistics, Hacettepe University, Ankara, Turkey; ^4^Department of Preventive, Restorative and Pediatric Dentistry, University of Bern, Bern, Switzerland

## Abstract

The purpose of this study was to assess the clinical performance of composite restorations placed with different adhesive systems in primary teeth. In 32 patients, 128 composite restorations were placed using a split-mouth design as follows (4 groups/patient): three-step etch-and-rinse (Group 1), two-step etch-and-rinse (Group 2), two-step self-etch (Group 3), and one-step self-etch (Group 4). The restorations were clinically evaluated at baseline and at 6, 18, and 36 months according to the FDI criteria. There was no significant difference between the adhesive systems in retention of the restorations (*p* > 0.05). Over time, there was a statistically significant decrease in marginal adaptation in all groups, whereas surface and marginal staining significantly increased in Groups 3 and 4 (*p* < 0.05). The etch-and-rinse adhesive systems resulted in better marginal adaptation than the self-etch adhesive systems (*p* < 0.05). It was concluded that preetching of the primary enamel might help improve the clinical performance of the self-etch adhesive systems in primary teeth.

## 1. Introduction 

Tooth-colored materials are widely used in pediatric dentistry for the restoration of carious primary teeth [1]. Of these materials, resin composites have been gaining increasing popularity over the past few decades because of their favorable esthetic and mechanical properties [2, 3].

Composite restorations are placed following pretreatment of the cavities with an adhesive system. Until recently, etch-and-rinse adhesive systems have been regarded as the gold standard owing to their good clinical and laboratory record [4]. However, these adhesive systems involve numerous application steps. In pediatric dentistry, and especially when treating uncooperative children, such a time-consuming technique is undesirable and oftentimes difficult to carry through [5]. To simplify the bonding procedure and to reduce technique sensitivity of the etch-and-rinse adhesive systems, self-etch adhesive systems have been developed [6]. For the treatment of primary teeth, the etch-and-rinse adhesive systems may offer the advantage of a separate acid etching step, which has been suggested to be necessary for the prismless enamel [7]. Compared to the permanent dentin, the inorganic content of intratubular primary dentin is less. The number of dentinal tubules is lower, resulting in less surface moisture and increased susceptibility of the primary dentin to decalcification [5, 8, 9]. In this regard, the self-etch adhesive systems may offer the advantage of limited decalcification of the primary dentin [10]. The number of clinical studies comparing etch-and-rinse adhesive systems with self-etch adhesive systems, however, is limited which means that the superiority of any one category of adhesive system in primary teeth has yet to be firmly established [11–13]. To the best of our knowledge, no study has yet compared the clinical efficacy of various types of etch-and-rinse or self-etch adhesive systems in primary teeth. Therefore, the aim of this clinical trial was to evaluate the clinical performance of composite restorations placed with one of four adhesive systems (a three-step etch-and-rinse, a two-step etch-and-rinse, a two-step self-etch, or a one-step self-etch adhesive system) in primary teeth. The null hypothesis to be tested was that there was no difference in the clinical performance of the adhesive systems after 3 years.

## 2. Methods

**Figure 1:**
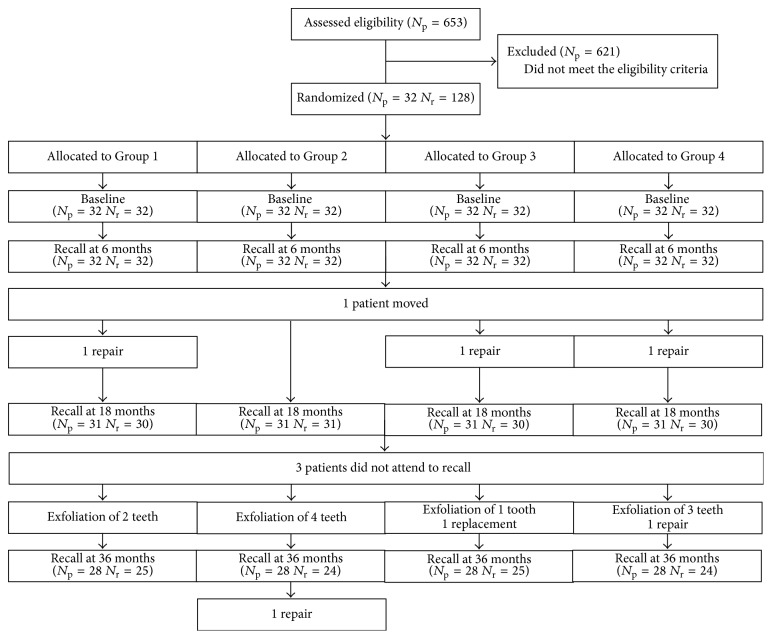
Flow diagram. *N*_p_: number of patients, *N*_r_: number of restorations.

The study was approved by the local ethics committee of the university (Project number FON 08/58). A total of 653 healthy children aged 4–7 years attending the Pediatric Dentistry Department were examined to determine their eligibility for the study. Thirty-two of the children were selected as they met the study criteria including the presence of four first and/or second primary molars with proximal caries extending into dentin and having occlusal and proximal contacts (Figure 1). The procedure, risks, possible discomforts, and benefits were explained to the parents and their informed consents were obtained prior to the study.

The four carious primary molars in each patient were randomly allocated into four groups. For randomization (beginning from the lower right quadrant followed by the lower left quadrant, upper left quadrant, and upper right quadrant), the four teeth in each patient were sorted. From the web site (https://www.random.org/), sequences for group numbers (1 to 4) were generated. The group numbers in each sequence and the teeth were matched.

The restorations were placed by one trained operator. Local anesthesia was administered, a rubber dam was placed, and nonbeveled Class II cavities were prepared on the primary teeth.

## 3. Restoration Groups

In Group 1, a three-step etch-and-rinse adhesive system (OptiBond FL, Kerr Corporation, Orange, CA, USA) was used. The cavity was etched with 37,5% phosphoric acid (30 s for enamel and 15 s for dentin), rinsed thoroughly for 15 s, and gently air-dried for 5 s. The primer was applied by light scrubbing motions for 15 s and then gently air-dried for 5 s. The adhesive was applied in a uniform layer and light-cured for 30 s.

In Group 2, a two-step etch-and-rinse adhesive system (XP Bond, Dentsply DeTrey, Konstanz, Germany) was used. The cavity was etched with 36% phosphoric acid for the same durations as in Group 1. The adhesive was applied, left undisturbed for 20 s, air-dried thoroughly for 5 s to evaporate the solvent, and light-cured for 20 s.

In Group 3, a two-step self-etch adhesive system (AdheSE, Ivoclar Vivadent, Schaan, Liechtenstein) was used. The primer was applied for 15 s and brushed for another 15 s, and excess primer was then dispersed with a strong air stream for 5 s. The bond was applied, dispersed with a weak air stream for 5 s, and light-cured for 10 s.

In Group 4, a one-step self-etch adhesive system (G-Bond GC Corporation, Tokyo, Japan) was used. The adhesive was applied, left undisturbed for 10 s, dried thoroughly under maximum air pressure for 5 s, and light-cured for 10 s.

Following treatment of the cavity with one of the four adhesive systems, a resin composite (Esthet-X, Dentsply DeTrey, Konstanz, Germany) was placed in 2 mm increments, each light-cured for 20 s with a visible light curing unit (Hilux Ultra Plus, Benlioglu Dental, Ankara, Turkey; light curing intensity of 700 mW/cm^2^). Finishing and polishing of the restorations were done with diamond finishing burs, yellow rubber cups (Diatech, Diatech Dental AG, Heerbrugg, Switzerland), and aluminum oxide discs (Sof-Lex, 3M/ESPE, Seefeld, Germany).

## 4. Clinical Assessment

All restorations were clinically evaluated at baseline (1 week after placement) and after 6, 18, and 36 months by one trained operator who was blinded to the restorative group under examination. Standard photographs of the restorations were taken and the assessment was made according to the FDI World Dental Federation criteria with codes ranging from 1 to 5 (Code 1: clinically excellent, Code 2: clinically good, after polishing very good, Code 3: clinically sufficient, Code 4: clinically unsatisfactory, repair is necessary, and Code 5: clinically poor, replacement is necessary) (Table 1) [14].

To determine the intraexaminer reliability, 20 restorations at different recalls were reevaluated with an interval of 1 week and statistically evaluated with Kappa test.

## 5. Radiographical Assessment

All restorations were radiographically evaluated at baseline and after 18 and 36 months by one trained operator who was blinded to the restorative group under examination. Radiographic examination was not performed at 6 months to lessen radiation exposure. The periapical radiographs were obtained with the parallel technique and scanned at 2400 dpi with a scanner (Epson Expression 10000 XL, Seiko Epson Co., Nagano, Japan). From these images, the tooth-restoration interface was analyzed at ×600 magnification using an image analysis program (ImageJ 1.43n, NIH, USA).

To determine the intraexaminer reliability, all radiographs were reevaluated after 1 week by the same operator. Intraexaminer reliability was analyzed by intraclass correlation coefficient (ICC).

## 6. Statistical Analyses

The statistical analyses were performed by a statistician. The data obtained were subjected to statistical analysis at a 0.05 level of significance. For each recall time, the groups were compared using the Kruskal-Wallis test (intergroup comparisons). The differences between recall times in each group were compared using the Friedman test (intragroup comparisons).

## 7. Results

**Figure 2:**
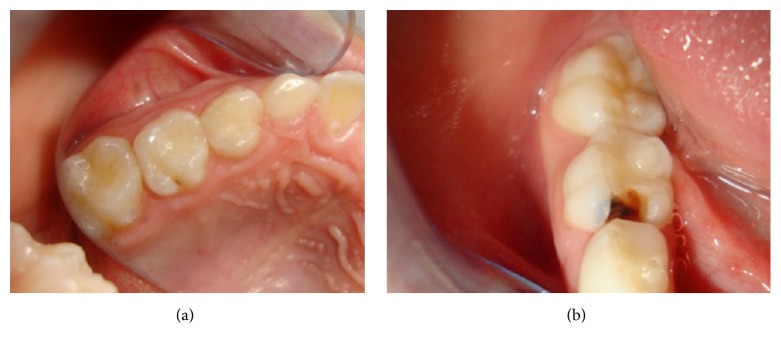
Clinically excellent occlusomesial restoration on upper second primary molar at 36 months (a); lost restoration on lower second primary molar (loss at 30 months) (b).

Of the 32 children (mean age 5.96 ± 0.82 years) who participated in the study, 27 (84.4%) were girls and 5 (15.6%) were boys. The intraexaminer reliability of the clinical and radiological assessments was 96% and 82%, respectively. Examples of a clinically excellent restoration and a lost restoration are shown in Figure 2.

The overall failure rates at 36 months were 3.8%, 4.2%, 7.4%, and 7.7% for Groups 1–4, respectively. The failures were due to partial restoration loss in 5 teeth (1 tooth in Groups 1, 2, and 3 and 2 teeth in Group 4) and total restoration loss in 1 tooth (Group 3, Table 3).

At the end of the study, all restorations had clinically excellent surface gloss. The rate of restorations with excellent color match and anatomic form varied from 92% to 100%. In the radiographic evaluation, many of the restorations were assigned a score of 2 at all recall appointments (Tables 2 and 3).

At 36 months, secondary caries was detected in 3 teeth (1 tooth in Group 2, 2 teeth in Group 3). In Group 4, secondary caries was detected in one patient at 18 months but the patient did not attend to further recall (Table 4).

In regard to proximal contact point criteria, many of the restorations were given a score of 4, because of physiological spaces in primary dentition (Table 3). Those restorations were not repaired and no clinical symptoms were detected during the evaluation period. Postoperative sensitivity, tooth fracture, localized soft tissue reactions, and oral and somatic psychiatric symptoms were not detected throughout the study (Table 4).

With respect to intragroup differences over time, statistically significant differences were found for the surface and marginal staining and for the marginal adaptation criteria (*p* < 0.05). Surface and marginal staining for the restorations in Groups 3 and 4 had increased significantly at 36 months compared to those recorded at baseline and at 6 months (*p* < 0.05). Marginal adaptation of restorations in Groups 1 and 2 had decreased significantly at 36 months compared to those at baseline and at 6 months (*p* < 0.05). Marginal adaptation of restorations in Group 3 had decreased significantly at 18 months and 36 months compared to those at baseline and at 6 months. Finally, marginal adaptation of restorations in Group 4 had decreased significantly at 18 months and 36 months compared to at baseline and at 36 months compared to 6 months (*p* < 0.05) (Tables 2 and 3).

Regarding intergroup differences, statistically significant differences were found for the marginal adaptation criterion (*p* < 0.05). At 6 months and at 18 months, there was a statistically significant difference between Group 1 and Group 3 and between Group 2 and Group 3. At 36 months, there was a statistically significant difference between Group 1 and Group 4 (*p* < 0.05) (Table 3).

## 8. Discussion

Split-mouth studies offer a way of comparing restorations intraindividually and offer the advantage of limiting patient-dependent variables [15]. The major disadvantage of split-mouth studies, however, is the difficulty in gathering patients with a sufficient number of similar types of caries lesions. The sample size of the present study (32 patients) may be regarded as small, but it reflects the great difficulty in finding four similar proximal caries lesions in primary teeth of one individual. The lack of split-mouth studies including four Class II restorations in primary teeth in the literature also is an indication of this difficulty.

Available studies on the clinical performance of restorative materials in primary teeth mainly focus on the efficacy of compomer, amalgam, and glass ionomer cement. A few studies have reported on the clinical success of resin composites in primary teeth [12, 15–20]. In the majority of these studies, the restorations were assessed according to the Ryge criteria. Only one retrospective and one prospective study used the FDI criteria, and these focused on survival of the restorations rather than giving a detailed analysis as was done in the present study [12, 21].

Hickel et al. [14] have proposed a system according to which the results of studies that used the Ryge criteria can be compared to those that used the FDI criteria. According to this system, the FDI scores of 1 and 2 correspond to a Ryge score of alpha, a FDI score of 3 corresponds to a Ryge score of bravo, and the FDI scores of 4 and 5 correspond to the Ryge scores of charlie and delta. Application of this conversion system to the present results gives the following ranges of alpha and bravo scores for the surface and marginal staining criterion of the four groups: 66.7–88% (alpha) and 8.3–33.3% (bravo). These results are in accordance with previously reported ranges of marginal discoloration of 66.7–81% for alpha and 14–31.6% for bravo according to the Ryge criteria [17, 18, 20].

As for marginal adaptation, the rates reported in literature are 36.8–92% for alpha and 5–63.2% for bravo scores, indicating a wide variation between the different studies [15, 17, 18, 20]. Compared to those of the aforementioned studies, the rate of alpha scores in the present study is lower (7.7–48%) whereas the rate of bravo is higher (50–88.5%), a finding that may be related to the longer evaluation time of the restorations in the present study. No previous study in primary teeth has used an evaluation time of 3 years, and it is reasonable to expect a decrease in the alpha grading after 3 years compared to that after 18 months or 2 years as reported in the aforementioned studies [15, 17, 19, 20, 22, 23].

The manufacturers of the two etch-and-rinse adhesive systems recommend etching of the enamel and dentin for 15 seconds regardless of whether the procedure is used in primary or permanent teeth. In the present study, an etching time of 30 seconds was used for enamel [24]. Although no consensus has been reached regarding the most appropriate etching time for primary enamel, it is generally accepted that a longer etching time is required owing to the presence of an outer prismless layer [25]. The dentin may be etched as well during the enamel etching process, frequently at the bottom of the proximal box in primary teeth where no enamel layer is left. This could lead to an overetching of the dentin and reduced bond strength of the restorations. However, the results of the present study demonstrated no such effect on the clinical retention of the restorations.

The morphological differences between primary enamel and dentin require different mechanisms of action. The pH of the self-etch adhesive systems should be sufficiently low to remove the smear layer but should also be sufficiently high so as not to cause excessive demineralization of the dentin substrate [5, 26]. It has been suggested that self-etch adhesive systems might be more suitable for primary teeth owing to their less aggressive etching of the less mineralized primary dentin [27, 28]. The pH of the two-step and one-step self-etch adhesives used in the current study was 1.7 and 2.3, respectively [29, 30], thus classifying them as mild adhesive systems [29]. In the present study, the two etch-and-rinse adhesive systems resulted in better marginal adaptation and less marginal staining of the restorations than the two self-etch adhesive systems. This result may be attributed to the higher pH of the self-etch adhesive systems resulting in a shallow etching pattern in primary enamel and influencing the marginal integrity of the restorations [4, 31, 32]. In order to increase marginal integrity, preetching of the enamel has been suggested [33, 34]. However, contamination of the dentin with phosphoric acid is inevitable causing decalcification that is too deep to be completely infiltrated by the adhesive [35, 36]. The morphological differences between primary and permanent dentin mean that decalcification would be more pronounced on primary dentin [37]. In the present study, no preetching of the enamel was done. This decision was made for the aforementioned reasons as well as the aim to ascertain the absolute effect of the self-etch adhesive system on primary teeth. The fact that we found better surface and marginal adaptation and less marginal staining of the restorations treated with etch-and-rinse adhesive systems than self-etch adhesive systems may indicate the need for preetching primary enamel and for clinical studies in primary teeth looking into this aspect.

Numerous in vitro studies on primary teeth have found less marginal leakage and greater bond strengths with the etch-and-rinse adhesive systems as compared to self-etch adhesive systems [4, 38, 39]. Of the etch-and-rinse adhesive systems used in the present study, Ramires-Romito et al. [39] reported higher microtensile bond strength of primary enamel with OptiBond FL than with the self-etch version, OptiBond Solo SE. Lemos et al. [40] demonstrated higher bond strength and a better-defined etching pattern and resin tags on primary enamel with OptiBond FL than with OptiBond All-in-One. Despite the results reported for primary enamel, it has been suggested that optimal bonding to primary dentin is achieved with self-etch adhesive systems [4]. Nevertheless, there have been limited studies so far and the results regarding the survival of restorations made with etch-and-rinse versus self-etch adhesive systems in primary teeth are conflicting [12, 15].

There has been no other clinical study that tested the four current adhesive systems in primary teeth whereas studies with these adhesive systems in permanent teeth have been reported [41, 42]. Delbons et al. [41] reported no statistically significant differences among the four adhesive systems with respect to all parameters including retention, marginal adaptation, and staining. In a recent systemic review and a meta-analysis, it was concluded that besides adhesive strategy (i.e., etch-and-rinse versus self-etch), the specific brand is also important as there is a wide variation in the performance of adhesive systems [43, 44]. The present study, however, did find differences between etch-and-rinse adhesive systems and self-etch adhesive systems. Similar to our results, Perdigão et al. [42] reported more enamel marginal deficiencies for the self-etch adhesive systems and similar retention rates for etch-and rinse and self-etch adhesive systems.

Not only the adhesive systems but many other factors determine the durability of restorations in primary teeth. The failure rates for composite restorations vary between 13.6% and 22.5% in primary teeth [12, 15, 17, 21, 23]. The lower failure rates found in the present study may be related to the use of rubber dam, which has been found to be an important factor in achieving a higher survival rate of composite restorations [12]. It is also worth mentioning that all restored teeth had occlusal and proximal contacts, which could have been important for the survival of the restorations [14].

In the current study, many of the restorations were assigned a score of 4 for the proximal contact point criteria, implying the need for repair. According to the clinical experience of the present authors, many intact primary teeth are also given a score of 4 because of the physiological spaces in primary dentition. It was decided, therefore, not to repair those restorations, and no clinical symptoms were detected in the present study.

In addition to the aforementioned criteria, two modifications were made for the radiographic evaluation parameter. In the parameter, the steps and gaps between the tooth and restoration were classified from 1 to 5 according to their dimensions. In the FDI criteria, although positive/negative steps <150 *μ*m were scored as 2, gap dimensions <150 *μ*m were not included. It was learned that the reason of it was due to inadequate resolution of the radiographs that may prevent proper radiographic analysis (correspondence with Dr. R. Hickel). In the present study, the score 2 included gaps <150 *μ*m as the resolution of the radiographs was adequate for analysis. Three restorations were detected with a gap size >250 *μ*m, implying the need for repair according to the radiographic evaluation parameter of the FDI criteria. Those restorations were not repaired or replaced because there has been no report about the gap dimensions causing clinical problems in primary teeth. No clinical symptoms were detected during the clinical follow-up period in the current research.

In the current research, the clinical performance of composite restorations was evaluated using the FDI criteria. The null hypothesis was rejected, as better marginal adaptation and less surface and marginal staining were found in restorations placed using etch-and-rinse adhesive systems than self-etch adhesive systems.

## 9. Conclusions

Under the limitations of this study, the following conclusions can be drawn:

1. In the current study, better marginal adaptation was found in composite restorations made with etch-and-rinse adhesive systems than with self-etch adhesives.
2. Marginal staining tended to increase over time in restorations made with self-etch adhesives.

## Figures and Tables

**Table 1:** The FDI evaluation criteria used to assess the restorations.

Esthetic properties	Surface gloss and roughness	Surface and marginal staining	Color match and translucency	Anatomic form

(1) *Clinically excellent*	(1.1) Comparable to enamel	(2.1) No marginal/surface staining	(3.1) Excellent match with the surrounding enamel	(4.1) Ideal anatomic form
(2) *Clinically good* (very good after polishing)	(1.2) Slightly dull, not noticeable from speaking distance	(2.2) Minor staining, easily removable	(3.2) Minor deviations in shade between tooth and restoration	(4.2) Form deviates slightly from the remainder of the tooth
(3) *Clinically sufficient* (no unacceptable effects but not adjustable without damage to the tooth)	(1.3) Dull surface, acceptable if covered with film of saliva	(2.3) Moderate staining not noticeable from a speaking distance, also present on other teeth	(3.3) Clear deviation but acceptable and does not affect esthetics	(4.3) Form differs but is not esthetically displeasing
(4) *Clinically unsatisfactory* (repairable)	(1.4) Rough surface, cannot be masked by saliva film, simple polishing is not sufficient	(2.4) Surface staining recognizable from speaking distance or severe localized marginal staining not removable by polishing	(3.4) Color and/or translucency are clinically unsatisfactory, recognizable from speaking distance	(4.4) Anatomic form is altered; the esthetic result is unacceptable
(5) *Clinically poor* (replacement necessary)	(1.5) Quite rough, unacceptable plaque retentive surface	(2.5) Severe surface staining or generalized and profound marginal discoloration	(3.5) Color match and/or translucency are clinically unsatisfactory	(4.5) Anatomic form is unsatisfactory and/or lost

Functional properties	Fracture of restorative material	Marginal adaptation	Proximal contact point	Radiographic examination

(1) *Clinically excellent*	(5.1) Restoration retained, no fractures, cracks, or chipping	(6.1) Harmonious outline, no gaps, no discoloration	(8.1) Normal contact point (dental floss can be inserted but not 50 *μ*m blade)	(9.1) No pathology, harmonious transition between restoration and tooth
(2) *Clinically good* (very good after polishing)	(5.2) Small hairline crack	(6.2) Small marginal chip fracture, can be eliminated by polishing	(8.2) Slightly too strong but acceptable. Floss can only be passed with force	(9.2) Small visible but acceptable excess and/or a positive/negative step or gap <150 *μ*m
(3) *Clinically sufficient* (no unacceptable effects but not adjustable without damage to the tooth)	(5.3) Two or more or larger hairline cracks and/or chipping (not affecting the marginal integrity or proximal contact)	(6.3) Gap <250 *μ*m, easily perceptible with a blunt explorer with a tip diameter of 250 *μ*m. Several small marginal fractures cannot be modified without damage and are unlikely to cause long-term effects	(8.3) Slightly too weak (50 *μ*m metal blade can pass easily whereas 100 *μ*m [two blades] cannot)	(9.3) Gaps and/or positive/negative step <250 *μ*m
(4) *Clinically unsatisfactory* (repairable)	(5.4) Chipping fractures affect marginal quality and/or proximal contacts; bulk fractures with or without partial loss of (<1/2 of the restoration)	(6.4) Gap > 250 *μ*m, may result in exposure of dentine or base	(8.4) Too weak (two 50 *μ*m metal blades can pass) and possible damage (food impaction).	(9.4) Gaps and/or positive/negative step >250 *μ*m and/or marked interradicular excess material
(5) *Clinically poor* (replacement necessary)	(5.5) (Partial or complete) loss of the restoration	(6.5) Restoration is loose but in situ	(8.5) Too weak and/or clear damage (food impaction) and/or pain gingivitis	(9.5) Gaps >500 *μ*m and/or secondary caries or apical pathology, tooth/restoration fracture

Biological properties	Postoperative sensitivity and tooth vitality	Secondary caries	Tooth cracks and fractures	Localized reactions of soft tissue in direct contact with the restoration

(1) *Clinically excellent*	(11.1) No hypersensitivity, normal vitality	(12.1) No secondary or primary caries	(13.1) Complete integrity	(15.1) Healthy mucosa adjacent to restoration
(2) *Clinically good* (very good after polishing)	(11.2) Low hypersensitivity for a limited period of time, normal vitality	(12.2) Very small, localized demineralization area	(13.2) Minor marginal crack or a hairline crack which cannot be probed. The patient has no clinical symptoms	(15.2) Healthy after minor removal of mechanical irritations (sharp edges, etc.)
(3) *Clinically sufficient* (no unacceptable effects but not adjustable without damage to the tooth)	(11.3) Premature/slightly more intense or delayed/weak hypersensitivity. No subjective complaints	(12.3) Larger areas of demineralization, preventive measures necessary (dentine not exposed)	(13.3) Enamel split or crack <250 *μ*m. No adverse effects	(15.3) Alteration of mucosa but no suspicion of causal relationship with filling material
(4) *Clinically unsatisfactory* (repairable)	(11.4) Premature/very intense or extremely delayed/weak hypersensitivity with subjective complaints or negative sensitivity	(12.4) Caries with cavitation	(13.4) Major enamel split gap > 250 *μ*m or dentine/base exposed or crack > 250 *μ*m (explorer penetrates)	(15.4) Suspected mild allergic, lichenoid, or toxicological reaction
(5) *Clinically poor* (replacement necessary)	(11.5) Very intense, acute pulpitis or nonvital tooth. Removal of restoration with or without immediate root canal treatment is required or the tooth must be extracted	(12.5) Deep secondary caries or exposed dentine that is not accessible for repair	(13.5) Cusp or tooth fracture	(15.5) Suspected severe allergic, lichenoid, or toxicological reaction

**Table 2:** Esthetic properties of the groups.

Criteria	Time	Score	Group 1	Group 2	Group 3	Group 4
			*n* (%)	*n* (%)	*n* (%)	*n* (%)
Surface gloss and roughness	Baseline	1	32 (100)	32 (100)	32 (100)	32 (100)
	6 months	1	32 (100)	32 (100)	32 (100)	32 (100)
	18 months	1	30 (100)	31 (100)	30 (100)	30 (100)
	36 months	1	25 (100)	24 (100)	25 (100)	24 (100)

Surface and marginal staining^*∗*^	Baseline	1	32 (100)	32 (100)	32 (100)	30 (93.8)
		3				2 (6.3)
	6 months	1	32 (100)	31 (96.9)	31 (96.9)	30 (93.8)
		3		1 (3.1)	1 (3.1)	2 (6.3)
	18 months	1	29 (96.7)	29 (93.5)	27 (90)	26 (86.7)
		2		1 (3.2)		
		3	1 (3.3)	1 (3.2)	3 (10)	4 (13.3)
	36 months	1	22 (88)	21 (87.5)	17 (68)	16 (66.7)
		3	3 (12)	2 (8.3)	8 (32)	8 (33.3)
		4		1 (4.2)^*∗∗*^		

Color match and translucency	Baseline	1	31 (96.9)	32 (100)	31 (96.9)	31 (96.9)
		2	1 (3.1)		1 (3.1)	1 (3.1)
	6 months	1	31 (96.9)	32 (100)	31 (96.9)	30 (93.8)
		2	1 (3.1)		1 (3.1)	2 (6.3)
	18 months	1	30 (100)	31 (100)	29 (96.7)	28 (93.3)
		2			1 (3.3)	2 (6.7)
	36 months	1	25 (100)	23 (95.8)	23 (92)	23 (95.8)
		2			1 (4)	1 (4.2)
		3		1 (4.2)	1 (4)	

Anatomic form	Baseline	1	32 (100)	32 (100)	32 (100)	32 (100)
	6 months	1	32 (100)	32 (100)	31 (96.9)	32 (100)
		4			1 (3.1)	
	18 months	1	30 (100)	31 (100)	30 (96.8)	30 (100)
		4			1 (3.2)	
	36 months	1	25 (100)	23 (95.8)	24 (92.3)	24 (100)
		3			1 (3.8)	
		4		1 (4.2)^*∗∗*^	1 (3.8)^*∗∗*^	

^*∗*^Significant changes in surface and marginal staining in Groups 3 (baseline/36 months *p* = 0.01, 6 months/36 months *p* = 0.02) and 4 (baseline/36 months *p* = 0.003, 6 months/36 months *p* = 0.03). ^*∗∗*^Due to partial restoration fracture.

**Table 3:** Functional properties of the groups.

Criteria	Time	Score	Group 1	Group 2	Group 3	Group 4
			*n* (%)	*n* (%)	*n* (%)	*n* (%)
Fracture of restorative material	Baseline	1	32 (100)	32 (100)	32 (100)	32 (100)
	6 months	1	30 (93.8)	32 (100)	31 (96.9)	31 (96.9)
		3	1 (3.1)			
		4	1 (3.1)		1 (3.1)	1 (3.1)
	18 months	1	29 (93.5)	30 (96.8)	30 (96.8)	29 (93.5)
		3	1 (3.2)	1 (3.2)		
		4	1 (3.2)		1 (3.2)	2 (6.5)
	36 months	1	24 (92.3)	23 (95.8)	24 (88.9)	24 (92.3)
		3	1 (3.8)		1 (3.7)	
		4	1 (3.8)	1 (4.2)	1 (3.7)	2 (7.7)
		5			1 (3.7)	

Marginal adaptation^*∗*^	Baseline	1	32 (100)	32 (100)	31 (96.9)	31 (96.9)
		3			1 (3.1)	1 (3.1)
	6 months^*∗∗*^	1	28 (87.5)	27 (84.4)	13 (40.6)	20 (62.5)
		2	2 (6.3)	2 (6.3)	9 (28.1)	3 (9.4)
		3	2 (6.3)	3 (9.4)	9 (28.1)	8 (25)
		4			1 (3.1)	1 (3.1)
	18 months^*∗∗∗*^	1	20 (66.7)	20 (64.5)	4 (12.9)	10 (32.3)
		2				1 (3.2)
		3	10 (33.3)	11 (35.5)	26 (83.9)	18 (58.1)
		4			1 (3.2)	2 (6.5)
	36 months^*∗∗∗∗*^	1	12 (48)	11 (45.8)	2 (7.7)	2 (7.7)
		3	13 (52)	12 (50)	23 (88.5)	22 (84.6)
		4		1 (4.2)^*∗∗∗∗∗*^	1 (3.8)^*∗∗∗∗∗*^	2 (7.7)^*∗∗∗∗∗*^

Proximal contact point	Baseline	3	15 (46.9)	14 (43.8)	16 (50)	13 (40.6)
		4	17 (53.1)	18 (56.3)	16 (50)	19 (59.4)
	6 months	3	15 (46.9)	20 (62.5)	19 (59.4)	13 (40.6)
		4	17 (53.1)	12 (37.5)	13 (40.6)	19 (59.4)
	18 months	2	1 (3.3)			1 (3.3)
		3	15 (50)	18 (58.1)	20 (66.7)	14 (46.7)
		4	14 (46.7)	13 (41.9)	10 (33.3)	15 (50)
	36 months	0	1 (4)	1 (4.2)	3 (12)	
		2	1 (4)			1 (4.2)
		3	10 (40)	8 (33.3)	12 (48)	9 (37.5)
		4	13 (52)	15 (62.5)	10 (40)	14 (58.3)

Radiographic examination	Baseline	2	31 (96.9)	31 (96.9)	30 (93.8)	31 (96.9)
		3		1 (3.1)	1 (3.1)	
		4	1 (3.1)		1 (3.1)	1 (3.1)
	18 months	2	29 (96.7)	30 (96.8)	29 (96.7)	29 (96.7)
		3		1 (3.2)		
		4	1 (3.3)		1 (3.3)	1 (3.3)
	36 months	0		1 (4.3)		1 (4.3)
		2	23 (95.8)	21 (91.3)	22 (91.7)	21 (91.3)
		3		1 (4.3)	1 (4.2)	
		4	1 (4.2)		1 (4.2)	1 (4.3)

^*∗*^Significant changes in marginal adaptation in Group 1 (baseline/36 months *p* = 0.000, 6 months/36 months *p* = 0.003); Group 2 (baseline/36 months *p* = 0.000, 6 months/36 months *p* = 0.001); Group 3 (baseline/18 months *p* = 0.000, baseline/36 months *p* = 0.000, 6 months/18 months *p* = 0.022, and 6 months/36 months *p* = 0.000); Group 4 (baseline/18 months *p* = 0.000, baseline/36 months *p* = 0.000, and 6 months/36 months *p* = 0.000). ^*∗∗*^Significant differences between Groups 1 and 3 (*p* = 0.008) and 2 and 3 (*p* = 0.017) at 6 months, ^*∗∗∗*^Groups 1 and 3 (*p* = 0.001) and 2 and 3 (*p* = 0.002) at 18 months, and ^*∗∗∗∗*^Groups 1 and 4 (*p* = 0.032) at 36 months. ^*∗∗∗∗∗*^Due to partial restoration fracture.

**Table 4:** Biological properties of the groups.

Criteria	Time	Score	Group 1	Group 2	Group 3	Group 4
			*n* (%)	*n* (%)	*n* (%)	*n* (%)
Postoperative sensitivity and tooth vitality	Baseline	1	32 (100)	32 (100)	32 (100)	32 (100)
	6 months	1	32 (100)	32 (100)	32 (100)	32 (100)
	18 months	1	30 (100)	31 (100)	30 (100)	30 (100)
	36 months	1	25 (100)	24 (100)	25 (100)	24 (100)

Secondary caries	Baseline	1	32 (100)	32 (100)	32 (100)	32 (100)
	6 months	1	31 (96.9)	32 (100)	32 (100)	32 (100)
		3	1 (3.1)			
	18 months	1	30 (100)	30 (96.8)	29 (96.7)	29 (96.7)
		2			1 (3.3)	
		3		1 (3.2)		1 (3.3)
	36 months	1	25 (100)	23 (95.8)	23 (92)	24 (100)
		2			1 (4)	
		3		1 (4.2)	1 (4)	

Tooth cracks and fractures	Baseline	1	32 (100)	32 (100)	32 (100)	32 (100)
	6 months	1	32 (100)	32 (100)	32 (100)	32 (100)
	18 months	1	30 (100)	31 (100)	30 (100)	30 (100)
	36 months	1	25 (100)	24 (100)	25 (100)	24 (100)

Localized reactions of soft tissue in direct contact with the restoration	Baseline	1	32 (100)	32 (100)	32 (100)	32 (100)
	6 months	1	32 (100)	32 (100)	32 (100)	32 (100)
	18 months	1	30 (100)	31 (100)	30 (100)	30 (100)
	36 months	1	25 (100)	24 (100)	25 (100)	24 (100)

Oral and somatic/psychiatric symptoms	Baseline	1	32 (100)	32 (100)	32 (100)	32 (100)
	6 months	1	32 (100)	32 (100)	32 (100)	32 (100)
	18 months	1	30 (100)	31 (100)	30 (100)	30 (100)
	36 months	1	25 (100)	24 (100)	25 (100)	24 (100)
